# Differential Transcriptomic Regulation in Sweet Orange Fruit (*Citrus sinensis* L. Osbeck) Following Dehydration and Rehydration Conditions Leading to Peel Damage

**DOI:** 10.3389/fpls.2021.732821

**Published:** 2021-08-31

**Authors:** Paco Romero, Maria Teresa Lafuente, Fernando Alferez

**Affiliations:** ^1^Department of Food Biotechnology, Institute of Agrochemistry and Food Technology-Consejo Superior de Investigaciones Cientificas (IATA-CSIC), Valencia, Spain; ^2^Horticultural Sciences Department, Southwest Florida Research and Education Center, University of Florida, Institute of Food and Agricultural Sciences, Immokalee, FL, United States

**Keywords:** citrus, fruit quality, non-chilling peel pitting, peel disorders, water stress

## Abstract

Water stress is the most important environmental agent that contributes to the crop productivity and quality losses globally. In citrus, water stress is the main driver of the fruit peel disorders that impact the quality and market ability. An increasingly present post-harvest peel disorder is non-chilling peel pitting (NCPP). Non-chilling peel pitting is manifested as collapsed areas of flavedo randomly scattered on the fruit and its incidence increases due to abrupt increases in the environmental relative humidity (RH) during post-harvest fruit manipulation. In this study, we have used a custom-made cDNA microarray containing 44k unigenes from *Citrus sinensis* (L. Osbeck), covering for the first time the whole genome from this species, to study transcriptomic responses of mature citrus fruit to water stress. In the study, the global gene expression profiles of flavedo from Navelate oranges subjected to severe water stress are compared with those fruits subjected to rehydration stress provoked by changes in the RH during post-harvest, which enhances the development of NCPP. The study results show that NCPP is a complex physiological process that shares molecular responses with those from prolonged dehydration in fruit, but the damage associated with NCPP may be explained by unique features of rehydration stress at the molecular level, such as membrane disorganization, cell wall modification, and proteolysis.

## Introduction

Water stress is the most important environmental agent causing crop productivity and quality losses (Boyer, 1982; Bray, 1997; Seki et al., 2007; Deluc et al., 2009; Rizzini et al., 2009; Alferez et al., 2020) and its relevance is increasing as the global climate is changing (MacCrackken, 2008). Indeed, changes in the precipitation patterns, runoff, and evapotranspiration fluxes, among other effects of climate change, have been already observed, and their incidence on crop yield and quality is expected to increase (Mancosu et al., 2015). In citrus fruits, water stress in the orchard and during fruit manipulation is the main cause not only of yield losses due to physiological alterations, such as pre-harvest fruit drop and leaf abscission (Gomez-Cadenas et al., 1996; Iglesias et al., 2007) but also of fruit peel disorders that impact the quality and marketability as well. The latter include stem-end rind breakdown (SERB) and post-harvest non-chilling peel pitting (NCPP). The SERB is due to prolonged dehydration (Albrigo, 1972) and manifests as collapsed areas of flavedo around fruit Calix, whereas NCPP is manifested as collapsed areas of flavedo randomly scattered on the fruit, and its incidence increases due to abrupt increases in the environmental relative humidity (RH) during post-harvest fruit manipulation (Alférez et al., 2003). This disorder may also occur in the harvested citrus fruits exposed to mild dehydration (Alferez et al., 2005; Romero et al., 2012), and in the harvested fruits held under saturated RH (Establés-Ortiz et al., 2016; Romero et al., 2020a), although the peel damage severity is lower than that occurring following rehydration of dehydrated fruit. These problems are becoming increasingly important in the past years, due to a combination of factors, such as the deficit of water resources in most of the citrus growing regions, the distance from orchards to packinghouses, and the increased time of fruit standby before packing line processing due to the production needs and market constraints (Cronje et al., 2017). Indeed, although the general climate conditions may vary among the citrus growing regions, there are common environmental factors during the harvesting seasons that affect water status in fruits leading to stress and peel disorders. Those show a similar pattern in RH that includes sharp variations in RH during the harvest season of susceptible varieties or periods of prolonged dehydration prior to post-harvest handling. For example, in Valencia (Spain), NCPP is likely to develop in Navelate and Navelina oranges after several days of sustained dry winds followed by humid winds from the Mediterranean (Agusti et al., 2001; Alférez et al., 2003); Navelate oranges grown in the Mediterranean climatic areas of South Africa are more prone to develop NCPP (Dr. P. Cronje, personal communication); finally, the pronounced fluctuations in RH are observed daily in early winter in Florida coincident with the harvesting season of the highly susceptible to NCPP Fallglo tangerine (Alferez et al., 2005). All these conditions have in common a rehydration stress event after tissue dehydration. Furthermore, rehydration of fruits dehydrated in the field is a common feature during post-harvest handling because of washing or the application of treatments to reduce decay, and also because of fruit storage at high RH.

Drought stress induces various molecular, biochemical, and physiological responses in plants (Yamaguchi-Shinozaki and Shinozaki, 2006; Seki et al., 2007; Oliver et al., 2020) and fruits (Deluc et al., 2009; Rizzini et al., 2009; Romero et al., 2012; Alferez et al., 2020). The major effects of water stress in plants are stomatal closure, inhibition of thylakoid mediated electron transport, membrane damage (Bohnert and Sheveleva, 1998), activation of antioxidant enzymes (Yuqing et al., 2021), and other diverse molecular responses, such as activation of signal transduction, lipid, and sugar metabolism, wax synthesis, cell wall regulation, and osmotic adjustment (Chen et al., 2020). In citrus plants, drought likewise induces the expression of key genes for amino acid metabolism, osmotic stress response proteins, reduction of reactive oxygen species, and transcription regulation (Gimeno et al., 2009). Previous study in our labs has shown that in citrus, the severity of water stress differentially affects the expression of phospholipase (PL)-encoding genes and the abscisic acid (ABA) regulation and/or response in both the fruit and the whole tree (Romero et al., 2013a, 2014; Alferez et al., 2020). Also, response to mild water stress in the fruit involves processes such as di, tri-valent inorganic cation transport, and molecular response to water deprivation which include both ABA-dependent and independent genes, as well as a non-impaired carbohydrate biosynthetic machinery (Romero et al., 2012). In addition, the deregulation of the ABA-signalosome components under mild water stress conditions has been linked with a higher susceptibility to NCPP development (Romero et al., 2013b). When considered together, available data up to date point out that the response and tolerance to water stress in citrus plants involve inhibition of proteolysis, activation of ABA signaling pathway and biosynthesis, phospholipid metabolism and signaling, and reinforcement of ribosomal structure (Romero et al., 2020a).

The dehydration and rehydration stresses involve disparate mechanisms at the biophysical level, for example, different evolution of turgor potential and its components across the anatomically different flavedo (the outer colored part of the fruit peel), external and internal albedo (the spongy white mesocarp) (Alférez et al., 2010), as well as different biochemical response (Alférez et al., 2008). Therefore, molecular responses to rehydration in fruit suffering previous dehydration or water stress should differ from those triggered by dehydration alone.

Previous research has shown that even slight variations in water potential may alter the synthesis of membrane lipids in different plant systems among other effects (Quartacci et al., 2002). Most of the research concerning dehydration and rehydration processes has been performed in leaves from desiccation-tolerant (resurrection) plants, with physiology adapted to withstand the sharp variations in water potential (Navari-Izzo et al., 2000; Benadjaoud et al., 2013). Some studies have been also performed in other crops such as papaya seedlings, showing changes in ABA levels in the roots and leaves during rewatering (Mahouachi et al., 2007). In the case of citrus plants, research on leaf abscission following rehydration after water stress has shown activation of several processes, such as cell wall modification (Agusti et al., 2012); it is also well-established that ABA accumulates in the roots in response to water deprivation and subsequent stress and that rewatering reduces ABA content (Gomez-Cadenas et al., 1996), illustrating the involvement of this hormone in the signaling of dehydration-rehydration processes. However, the regulatory networks involved in modulating the response to rehydration stress in citrus fruit are unknown.

Large-scale microarray analysis has been demonstrated to be a powerful tool to unravel complex regulatory networks involved in stress responses for years. In fruit, it can serve as a means to identify genes involved in the tolerance to the stresses that subsequently can be selected by breeding or used by the transformation approaches to obtain novel varieties with improved characteristics. In this study, we have used a custom-made cDNA microarray containing 44k unigenes from *Citrus sinensis* (L. Osbeck), which covers, for the first time, the whole genome from this species (Romero et al., 2020a). The main objective of the current study is to produce valuable information on the transcriptomic response of mature citrus fruit to water stress conditions by comparing global gene expression profiles of flavedo from Navelate oranges subjected to severe water stress (dehydration) with those fruits subjected to rehydration stress provoked by changes in RH during post-harvest, which enhances the development of NCPP, the main post-harvest peel disorder related to abiotic stress. The experimental conditions used in this study are not standard post-harvest practices for citrus fruit but intended to promote NCPP in controlled conditions that allow detailed study of this disorder, as shown in previous studies (Alférez et al., 2003). The data show unique features of rehydration stress that make it different from severe dehydration and suggest new mechanisms involved in the loss of fruit quality and marketability during post-harvest.

## Materials and Methods

### Plant Material and Post-harvest Treatments

**Figure 1:**
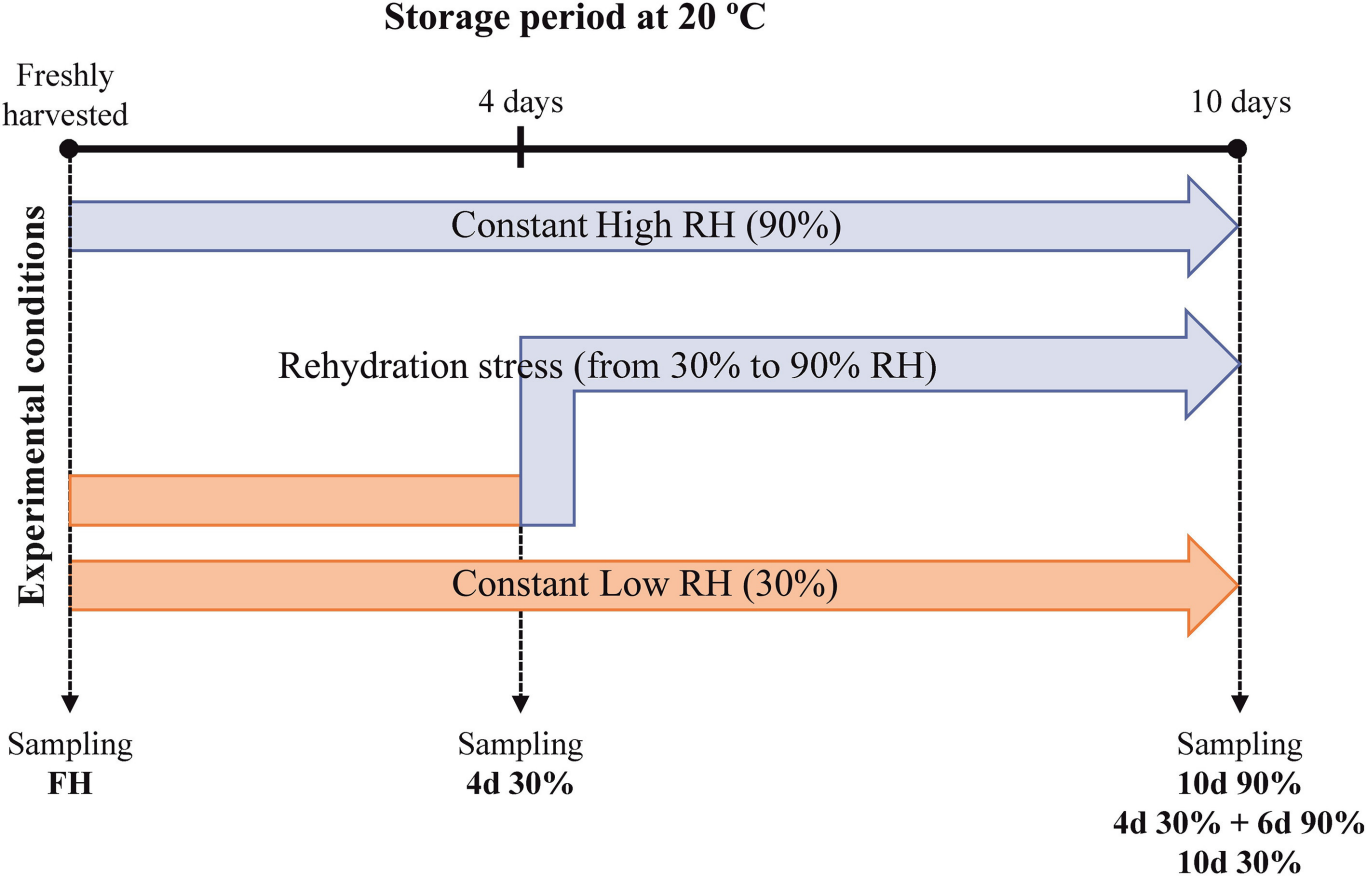
Schematic representation of the experimental design.

Full mature Navelate [*Citrus sinensis* (L. Osbeck)] sweet oranges were harvested from the adult trees grown in commercial groves in Lliria (Valencia, Spain). The fruits were immediately delivered to the laboratory, so they were not subjected to pre-harvest water stress before treatments and divided into three lots after selecting those fruits uniform in size and without visual defects or damages. One lot was stored continuously at low RH (30% RH) for 10 days; a second lot was stored at high RH (90% RH) for the same period (non-dehydrated fruits kept at constant RH); and the third lot was stored at 30% RH for 4 days prior to being transferred to 90% RH for 6 additional days (10 days from harvest) as the condition for studying the rehydration stress (Figure 1). All the treatment lots were stored at 20°C to effectively induce NCPP as previously described (Alférez et al., 2003). At each sampling period, flavedo samples were collected, frozen, and homogenized in liquid nitrogen, and kept at −80°C for later analysis. The four biological replicates per treatment, each consisting of five fruits, were collected at each sampling period and used for molecular analyses as described below.

For peel damage estimation, three biological replicates of 10 fruits each per treatment were used. The peel of the fruit was inspected for the intensity and extension of NCPP symptoms to rate on a scale from 0 (no pitting/damage) to 4 (severe pitting/damage). The severity index was calculated by adding up the products of the number of fruits in each category multiplied by its score and then dividing the total obtained by the number of fruits evaluated, as described by Lafuente et al. (1997). The same fruits were used at the various evaluation dates for each treatment.

### RNA Isolation and cDNA Synthesis

Total RNA was extracted from frozen flavedo samples as previously described by Romero et al. (2019). To remove genomic DNA contaminations, total RNA was treated with Ribonuclease-free DNase (Invitrogen) following the instructions of manufacturer. RNA integrity was evaluated by agarose gel electrophoresis and its concentration was determined spectrophotometrically with a NanoDrop ND-1000 spectrophotometer (Thermo Scientific, Wilmington, DE, USA). RNA quality was further verified with a Model 2100 Total RNA BioAnalyzer by using the Eukaryote Total RNA Nano kit (Agilent Technologies, Santa Clara, CA, USA). cDNAs from all the replicates and conditions were synthesized from 2 μg of RNA by using SuperScript III RT (Invitrogen, Waltham, MA, USA) in presence of Oligo(dT) 20-mer (Invitrogen) and Ribonuclease Inhibitor (Invitrogen), according to instructions from the manufacturer.

### cDNA Labeling, Microarray Hybridization, and Data Acquisition and Analysis

The transcriptomic changes occurring in the flavedo of Navelate oranges stored at 20°C and different RH conditions were determined. The four biological replicate samples per treatment and sampling period were analyzed with a custom-made *Citrus* cDNA microarray previously described by Romero et al. (2020a). This microarray was developed using the Agilent technology, contained 44k unigenes, and covered the whole *C. sinensis* genome.

cDNA labeling and microarray hybridizations were performed by following the recommended protocols of Agilent Technologies, as described in Romero et al. (2020a). The microarray hybridizations were performed according to the two-color protocol (Two-Color Microarray-Based Gene Expression Analysis v. 6.5, Agilent Technologies) and hybridized microarrays were scanned using a DNA Microarray Scanner (Model G2505C, Agilent Technologies, Santa Clara, CA, USA). Feature data were extracted with the Feature Extraction Software (Agilent Technologies) using the default settings. Under the R environment, bioconductor and Limma packages were used, respectively, for data preprocessing and analysis and for differential expression analysis. Data normalization within- and among-arrays was performed by following the Loess and Aquantile algorithms, respectively. The differential expressed genes (DEG) were identified by applying a false discovery rate (FDR) below 1% and a cutoff of Log_2_ Fold Change ≥1. Gene Ontology (GO) analysis was performed on the groups of DEG by using the GOStats package (Falcon and Gentleman, 2006), which allowed to identify of biological processes (BP), molecular functions (MF), and cellular components (CC) significantly (*p*-value < 0.01) under- or over-represented respect to the reference sample. In these analyses, only DEG showing at least a two-fold change in expression (Log_2_ Fold Change ≥ 1) were considered. Venn diagrams showing the DEG distribution were plotted by using Venny 2.1 tool (https://bioinfogp.cnb.csic.es/tools/venny). The repeatability and distribution of the experimental samples were verified in a principal component analysis (PCA) considering those DEG satisfying an ANOVA analysis (*P* < 0.01) for all the conditions represented in Venn diagrams by using the STHDA tools (http://www.sthda.com). Hierarchical clustering analysis (HCA) and heatmap representation were performed by using the Instant Clue software (Nolte et al., 2018). For HCA, Euclidian distance and complete linkage were applied to rows (genes) and columns (samples). To visualize transcriptomic changes on a metabolic map, gene expression data for specific comparisons were loaded into the MapMan tool (Thimm et al., 2004).

### Statistical Analyses

The significant differences between means of the NCPP incidence for each treatment were established after *post-hoc* Tukey's test (*P* ≤ 0.05) using Statgraphics (http://www.statpoint.net) webpage. Data are provided as the mean values ± SE of the different biological samples used in each treatment, as indicated in the figure legends.

## Results

### NCPP Development

**Figure 2:**
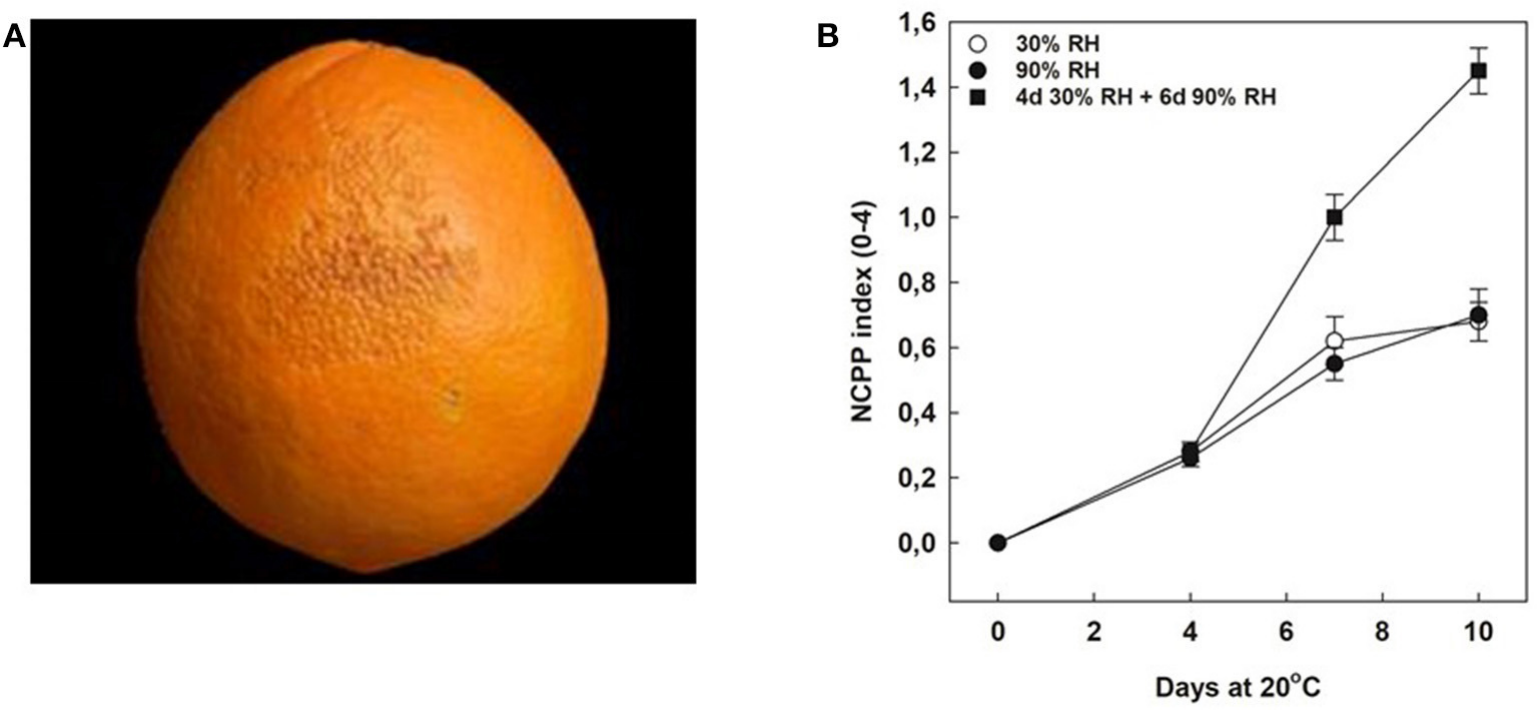
Effect of rehydration stress on peel damage development. **(A)** Typical symptoms of non-chilling peel pitting (NCPP) in mature Navelate oranges. **(B)** NCPP index in Navelate fruit stored for up to 10 days at 20°C and exposed continuously to 30% RH (white circles), or 90% RH (black circles), or for 4 days at 30% RH and then transferred to additional 6 days at 90% RH (squares). Values are means of three biological replicates of 10 fruit samples each per condition. Asterisks indicate statistical differences according to Tukey's test (*P* ≤ 0.05).

The incidence and development of NCPP were determined in full mature Navelate oranges stored for 10 days at 20°C under three different RH conditions: (1) constant low (30%) RH, (2) high (90%) RH, and (3) the abrupt changes in storage RH (from 30 to 90% RH). Under constant RH conditions, the development of NCPP was low (NCPP index of 0.6 in a rating scale from 0 to 4 by day 10), independently of the exposure of the fruits to low or high RH. In contrast, changing RH conditions by 4 days from low to high RH sharply increased NCPP severity during the following 6 days of storage at constant high RH. Thus, by 10 days, the NCPP index multiplied by 2.3 with respect to either constant low or high RH (Figure 2).

### Transcriptional Analysis of NCPP Development in Response to Rehydration Stress

The fruits from Navelate orange stored at constant high (10 days at 90% RH) or low (4 and 10 days at 30% RH) RH, as well as those being transferred from low to high RH (4 days at 30% + 6 days at 90% RH) were selected to compare changes in transcriptomic profiles with those occurring at freshly harvested (FH) fruits to identify the molecular mechanisms associated with the rehydration stress in citrus fruits and NCPP development.

**Figure 3:**
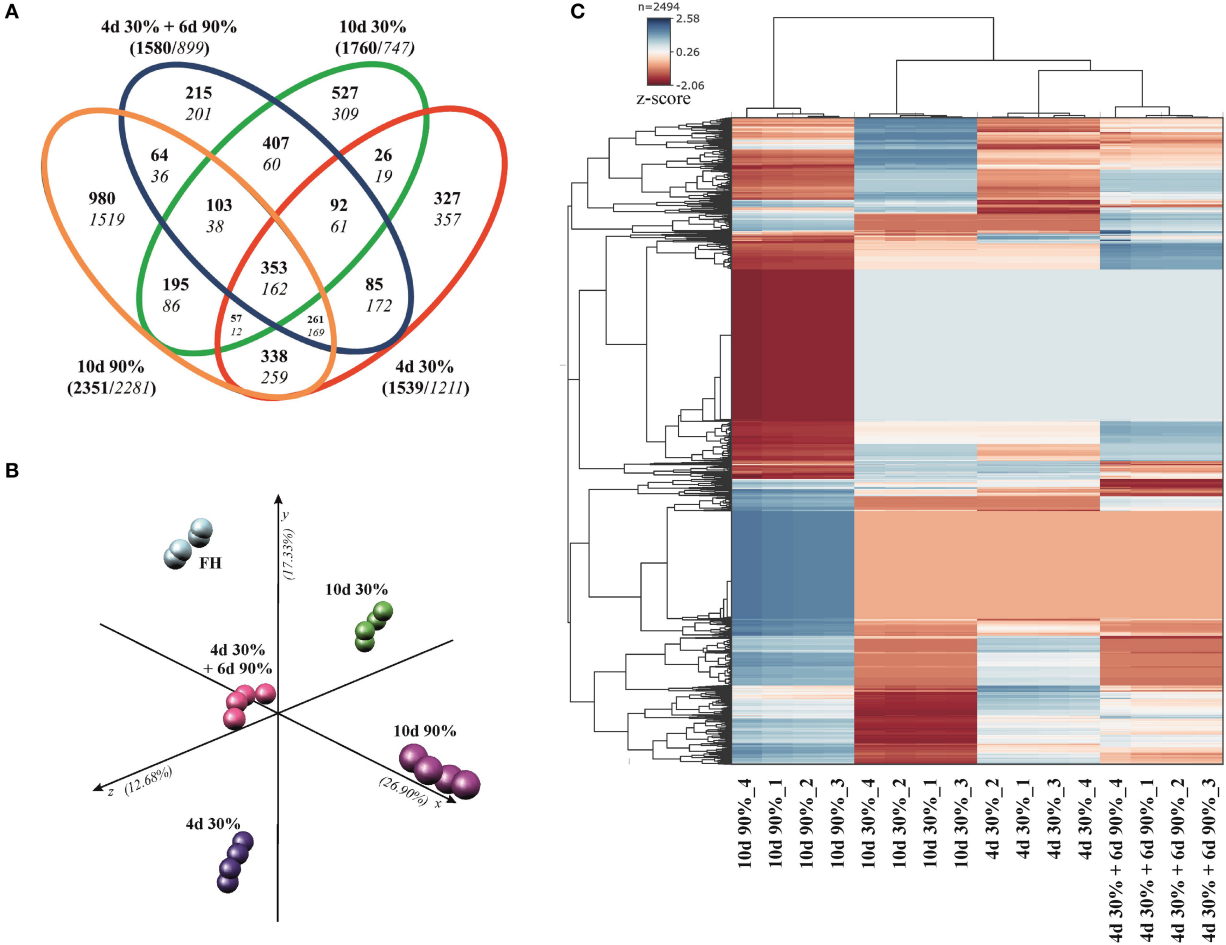
The differential expression and multivariate analysis in response to dehydration/rehydration stress. **(A)** Venn diagrams of Navelate fruit showing the distribution of differentially expressed genes (DEG, α = 0.05) satisfying a cutoff of Log_2_ FoldChange ≥1 for the comparisons between the fruit stored for 4 days at 30% RH, 10 days at 30% RH, 10 days at 90% RH, and 4 days at 30% RH + 6 days at 90% RH respect to the freshly harvested (FH) fruit. Inductions and repression are indicated in bold and italics, respectively, and the total number of induced and repressed DEGs for each conditions are indicated in brackets. **(B)** Principal component analysis (PCA), based on the DEG satisfying an ANOVA analysis (*P* < 0.01) for all conditions represented in the Venn diagrams. The three axes in PCA account for 56.91% of the total variance among conditions. **(C)** Hierarchical cluster analysis (HCA) and heatmap large-scale transcriptional profiles on the genes used for the PCA. Heatmap colors vary from dark blue (induction) to red (repression). Four biological replicates from each condition were used for all the analyses.

The DEGs obtained when comparing each condition vs. the FH fruit are listed in Supplementary Table 1. Venn diagrams in Figure 3A indicated that major changes in the number of DEG (FDR < 0.01, cutoff Log_2_FC ≥ ±1) occurred in fruit stored at high RH (10 days 90%, 4,632 DEG), followed by the fruit stored for a short (4 days 30%, 2,750 DEG) and long (10 days 30%, 2.507 DEG) period under low RH, and by the rehydration-stressed fruit (4 days 30% + 6 days 90%, 2,479 DEG). The number of upregulated genes was higher than that of downregulated in all conditions. Interestingly, the number of induced genes was about two-fold higher than that of repressed in fruit stored for 10 days under severe dehydration and in rehydration-stressed fruit; while in fruit stored for a shorter period (4 days) under 30% RH and in constant high RH fruit (10 days 90% RH), the number of up- and downregulated genes was similar.

The distribution of DEG in the Venn diagrams allows discriminating specific and common responses among all the conditions in the experimental design (Figure 3A). Fruit subjected to severe dehydration (30%) specifically upregulated 327 and 527 genes while repressed 357 and 309 genes, when stored for short (4 days) and long (10 days) periods, respectively. The number of DEG specifically regulated by transferring the fruit from low to high RH (rehydration stress) notably diminished (215 induced + 201 repressed), whereas it was more than three-fold higher (980 induced + 1,519 repressed) when fruit was held under constant high RH (10 days 90%). The number of commonly regulated DEGs between the rehydrated fruit and the rest of the conditions in the experimental design was the highest when compared with the long-term severe dehydration (10 days 30%, 407+60 DEG), followed by the short-term response to dehydration stress (4 days 30%, 85+172 DEG); this number was minimum when compared with the fruit stored under constant high RH (10 days 90%, 64+36 DEG). This analysis also revealed a set of genes commonly regulated by all the conditions (353+162 DEG) (Figure 3A).

The multivariate analyses (PCA and HCA) were performed to validate the repeatability of the data across replications and to the cluster samples according to their transcriptomic profiles. Under all conditions, the gene expression profiles of the four replicate samples were tightly clustered together (Figure 3B). Principal component analysis also revealed marked differences in gene regulation among the FH fruit and those following different storage conditions. The distance among samples stored at different RH was high (X-axis explaining the 26.90% of the total variation), but the distance caused by the time of exposure to dehydration stress, or the rehydration stress *per se* was also significant (y- and z-axes explaining a 30.01% of the total variation) (Figure 3B). Hierarchical clustering analysis clustered the samples in two major branches, splitting the samples of the experimental design in those fruit samples stored under high RH and those subjected at some point to severe dehydration. Furthermore, the fruit samples exposed to rehydration stress were allocated closer to the samples stored for a short (4 days) than for a long (10 days) period under low RH (Figure 3C).

The functional categorization analyses on the DEGs represented in the Venn diagrams identified BP, MF, CC, and KEGG metabolic pathways significantly (*P* < 0.01) under- or over-represented in the different storage conditions with respect to FH fruit. Gene ontology analyses revealed that the molecular mechanisms underlying severe dehydration and rehydration stresses were widely diverse (Supplementary Tables 2–5). To better understand the differential responses to these stresses and their relation to the development of NCPP, these GO categories were grouped by the regulation patterns. Pattern 1 includes those GO terms that were induced or repressed specifically by rehydration stress and, therefore, putatively involved in the NCPP development. The results showed high diversity among these BP and MF (Supplementary Tables 2, 3). Among them, it is worth noticing the induction of the “response to desiccation” and the “calcium-mediated signaling,” as well as the repression of the “regulation of ethylene biosynthesis” BP (Supplementary Table 2). These terms relate to the regulation of the “inositol-polyphosphate 5-phosphatase activity” and the “water channel activity” MF (Supplementary Table 3) and the “integral to plasma membrane” CC (Supplementary Table 4) and “inositol phosphate metabolism” metabolic pathway (Supplementary Table 5). Other interesting responses in relation to the NCPP development and peel damage were the induction of oxidoreductase and oxidase activities and the repression of the cellulase activity MF (Supplementary Table 3). Within the context of this study, it should be also pointed out those GO categories commonly regulated by short-term exposure to severe dehydration and by the rehydration stress (4 days at 30% RH and 4 days 30% RH + 6 days 90% RH, respectively) but not by the long-term storage, independently on the RH. These responses cannot be ruled out as related to NCPP development and are included in pattern 2. Among them, the transmembrane ions transport, the salicylic acid, jasmonate and auxins metabolism, the catabolism of terpene, the calcium-dependent phospholipid binding, and the response to sugar stimulus merit to be mentioned (Supplementary Tables 2–5). The rest of the patterns are not related to NCPP development but to a common regulation between severe dehydration and rehydration stresses (pattern 3), to the early responses to low RH that can be reversed by high RH exposure (pattern 4), to short- and long-term response to dehydration (pattern 5), and to the detachment or senescence stresses (pattern 6) (Supplementary Tables 2–5).

**Figure 4:**
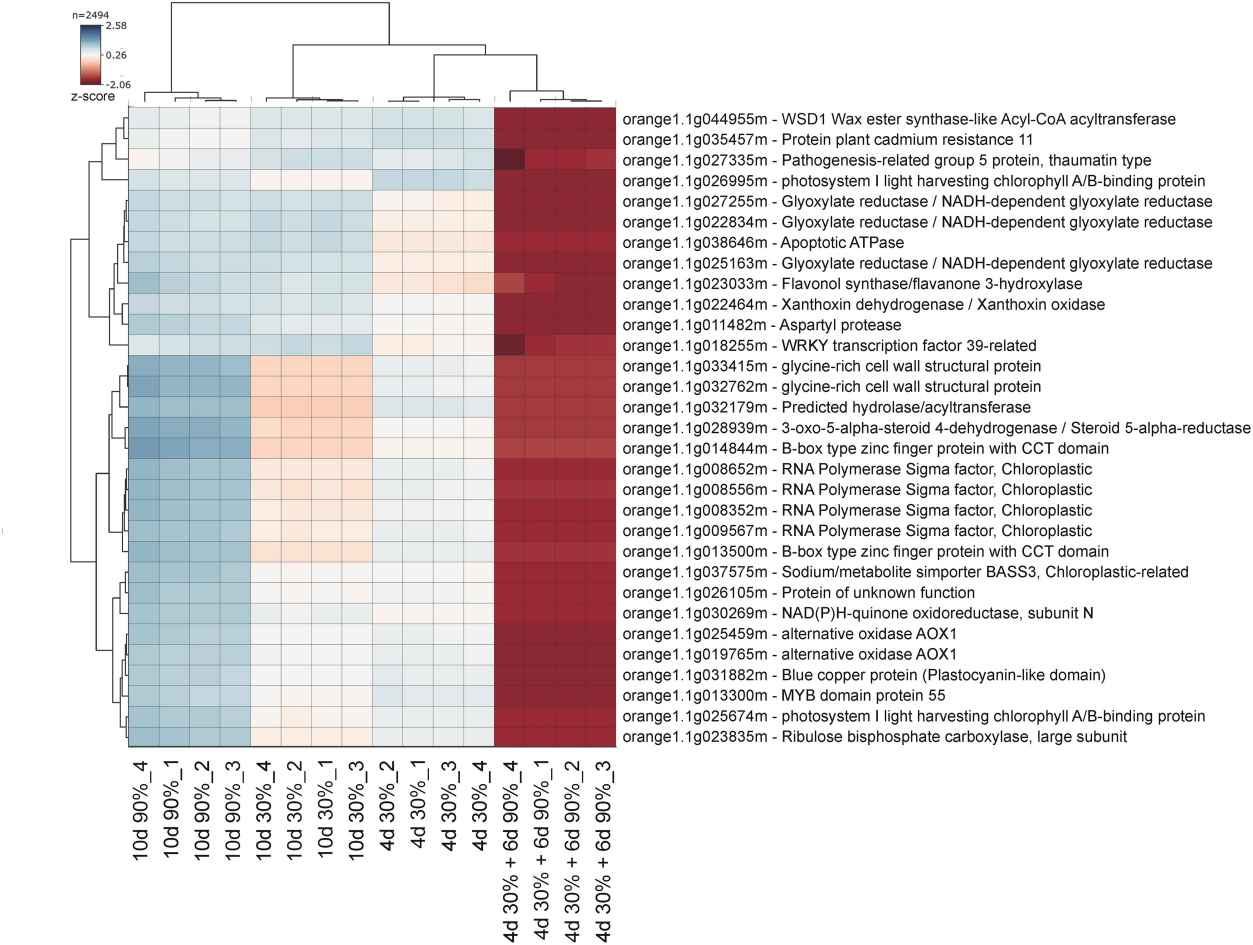
Gene clusters specifically repressed by rehydration stress. The genes included in the selected cluster from the heatmap large-scale transcriptional profiles are represented in Figure 3. Colors vary from dark blue (induction) to red (repression). Four biological replicates from each sampling condition were used for the analysis.

**Figure 5:**
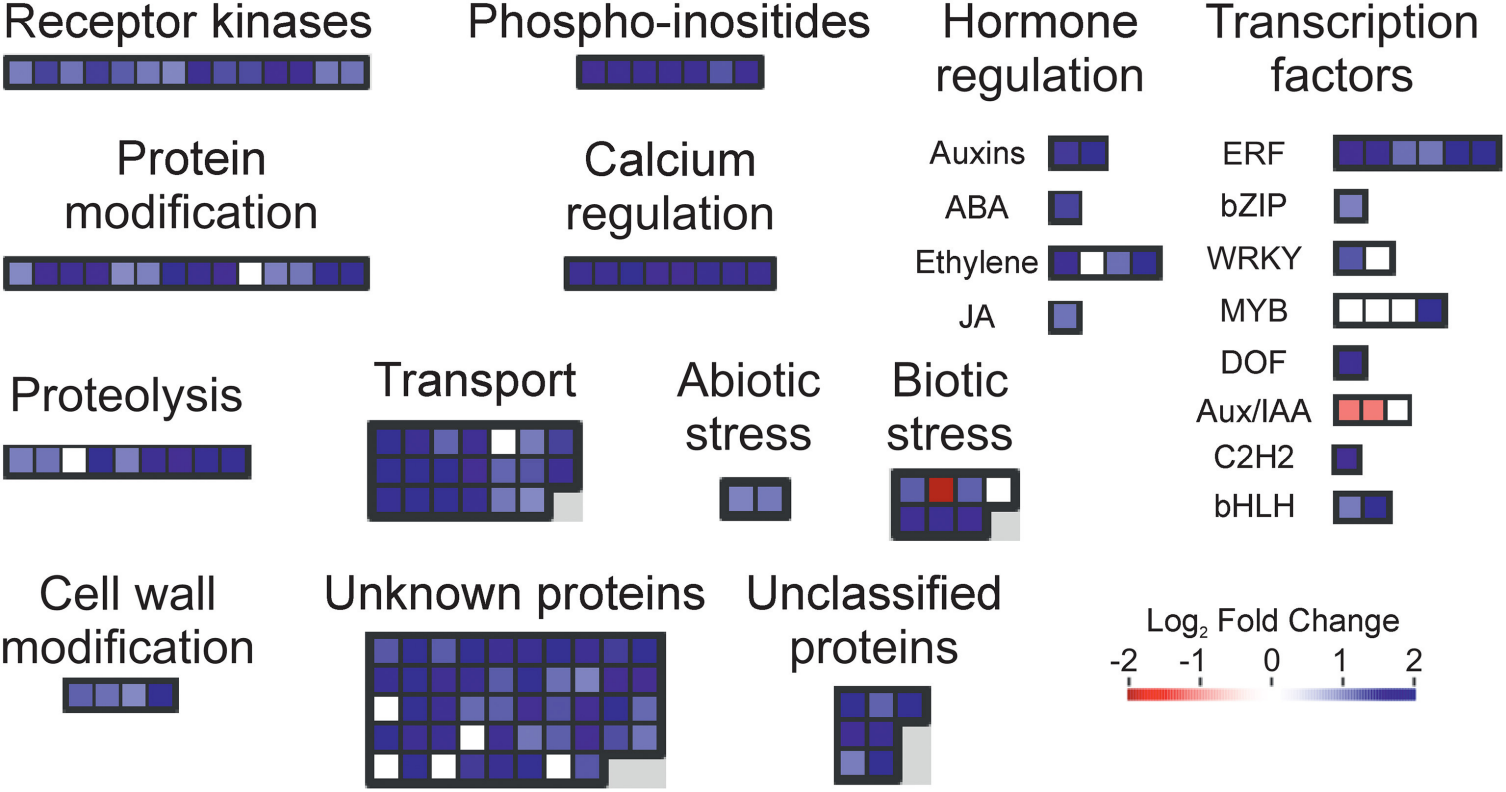
Metabolic overview changes caused by rehydration stress. Transcript accumulation comparison (Log_2_ FoldChange) in the flavedo of the fruit stored for 4 days at 30% RH + 6 days at 90% RH (rehydration-stressed fruit) with respect to the freshly harvested (FH) fruit. Red and blue squares represent the genes with decreasing and increasing transcript levels, respectively, in the stored fruit vs. the FH samples. The color scale is indicated in the figure.

To find a more specific response associated with the occurrence of peel damage after rehydration stress, we plotted the large-scale transcriptional profiles of all DEG between the storage conditions and the FH fruit (Figure 3C). This analysis provided a high number of expression patterns, which we then narrowed down to those showing a gene regulation specifically related to the rehydration stress condition. The patterns selected in this way then showed an opposite gene regulation between the fruit transferred from low to high RH (rehydration-stressed fruit) and the fruit stored under the rest of constant RH conditions. Within the expression pattern showing genes specifically repressed in response to rehydration stress, we found only 31 genes encoding for very diverse proteins (Figure 4). Among them, it is noticing the repression of genes related to photosynthesis and cellular energetics, cell wall proteins, and wax biosynthesis. However, no specific categories were obtained when a functional analysis was performed on this set of genes. The complementary expression pattern, however, included 195 genes that were induced only in response to rehydration stress. Gene Ontology analysis on this set of genes highlighted, among others, BP related to the response to biotic and abiotic stresses, the response to ABA and lipids, and the regulation of jasmonic acid signaling; in addition to the calcium ion binding MF, different stresses, such as osmotic stress, hypoxia, and the cell periphery CC (Table 1). To better visualize how the transcriptomic changes were distributed into a general overview and concrete metabolic pathways and how they are similar with the GO analysis, we performed a MapMan analysis (Figure 5). It is noteworthy that the group of unidentified proteins is highly upregulated, which may indicate processes related to NCPP development are still to unravel.

**Table 1:** Gene ontology (GO) categories related to NCPP development, specifically induced by rehydration stress according to the expression patterns shown in Figure 3.

**GO**	**Term**	**Number of genes**	**Fold enrichment**	**FDR**
**Biological Process**				
	Regulation of tryptophan metabolic process	2	200	0.025
	Response to chitin	9	19.68	0.000
	Jasmonic acid mediated signaling pathway	4	17.00	0.021
	Response to wounding	10	12.79	0.000
	Cellular response to hypoxia	6	7.74	0.026
	Response to lipid	15	5.58	0.000
	Response to abscisic acid	9	5.45	0.011
	Response to oxidative stress	8	5.27	0.025
	Response to other organism	17	4.69	0.000
	Response to osmotic stress	8	4.57	0.048
	Regulation of response to stimulus	10	4.04	0.029
	Defense response	12	3.81	0.015
	Regulation of transcription, DNA-templated	18	2.51	0.036
**Molecular Function**				
	Calcium ion binding	8	9.32	0.010
	Sequence-specific DNA binding	16	3.83	0.006
	DNA-binding transcription factor activity	18	3.30	0.008
**Cellular Component**				
	Cell periphery	28	2.11	0.041

## Discussion

In this study, we have used a customized microarray covering the whole genome from *C. sinensis* to study fruit flavedo responses to the rehydration stress during post-harvest. The abrupt changes in flavedo and albedo water potential and its components have been previously documented in response to the rehydration stress leading to NCPP. Even though weight gain in fruit was not recorded in response to an increase in RH storage conditions (environmental rehydration), fruit weight loss was immediately arrested following a change in RH, and water potential changed abruptly (data not shown), as reported elsewhere (Alférez et al., 2003, 2010; Cronje et al., 2017). The transcriptomic analysis indicates that changes in RH leading to severe NCPP during post-harvest manipulation of citrus fruit pose differentiated rehydration stress in contrast with the response to constant RH storage conditions. This study fills the gap of knowledge about the molecular mechanisms underlying abrupt changes in storage RH, which ends in the development of NCPP, a well-documented phenomenon in different citrus cultivars (Alférez et al., 2003; Alferez et al., 2005; Alférez et al., 2010; Cronje et al., 2017).

In the study, a storage experiment is performed in the conditions favoring the development of NCPP (Alférez et al., 2003) for 10 days. To gain insight into the molecular mechanisms specific to the NCPP development in response to this RH change, we analyzed the global transcriptomic response and compared it to non-stressed FH fruit (Table 1, Supplementary Tables 2–5).

We found sets of genes commonly regulated in all three conditions assayed (constant low RH, constant high RH, and change from low to high RH) when compared with FH fruit. These genes may be related to citrus fruit response to starvation stress caused by detachment, which has been related to NCPP in citrus fruit held at 90% RH (Cajuste et al., 2011; Establés-Ortiz et al., 2016; Romero et al., 2020a) or senescence, enhanced by keeping citrus fruit at low RH (Albrigo, 1972; Romero et al., 2013b). Among these common responses, sulfur metabolism appeared downregulated in all the conditions assayed. Sulfur metabolism occurs in the chloroplast, and both assimilation and metabolism are crucial for plant survival during stress (Rausch and Wachter, 2005). Also, the compounds involved in protein synthesis and defense contain sulfur (Lee et al., 2016), and thiol-containing compounds may be modulators of the stress response including oxidative stress (Khan et al., 2009; Szalai et al., 2009). Finally, the phenylpropanoid pathway, a well-known protective mechanism against diverse post-harvest stresses in citrus fruit aiming to content cell damage propagation (Lafuente et al., 2003; Cajuste and Lafuente, 2007) was activated in all three conditions.

An important finding of this study is that some molecular responses to short-term (4 days) dehydration were similar to those developed at long-term (10 days) dehydration but more similar to responses triggered by the change in RH. It could be possible that the similarity in these responses was due to a priming effect of the original dehydration conditions before changing RH, so no new expression patterns were developed upon provoking rehydration stress by changing RH storage conditions and/or that changing RH conditions resulted in only an aggravation of damage due to prolonged dehydration (Figure 1; Table 1). However, we ruled out the latter by comparing all the three stress conditions to FH fruit (Supplementary Tables 2–5). The nature of the responses to both short-term low RH and rehydration stress involved specifically transmembrane ions transport, salicylic acid, metabolism of jasmonate and auxins, catabolism of terpenes, calcium-dependent phospholipid binding, the response to osmotic stress, hypoxia, and the response to sugar stimulus (Supplementary Tables 2–5; Figure 5). Together, these responses can be related to loss of membrane integrity leading to NCPP, indicating a complex and orchestrated response to rehydration that ultimately leads to peel damage by energy metabolism and plasma membrane disorganization. The specific responses to rehydration stress also included an oxidative stress response, such as the activation of different oxidoreductase activities. Interestingly, some of these responses to rehydration stress that were also common to short-term dehydration seem to be related to cellular damage contention and hormonal regulation during rehydration and rewatering. The former includes a response to wounding, cell wall (downregulation of cellulase activity), and tryptophan metabolism. Some of these responses have been shown in citrus plants after a cycle of water stress and rehydration (Agusti et al., 2012). In other plants such as papaya, drought stress followed by rewatering induced changes in hormonal levels, specifically jasmonic acid (Mahouachi et al., 2007). In other plants, the role of salicylic acid in alleviating water stress and inducing antioxidant enzymes has been shown (Antonic et al., 2020; Wakchaure et al., 2020). In this system, rehydration stress following dehydration also involved the induction of gene expression related to jasmonic acid, salicylic acid, and antioxidative systems (Supplementary Table 2). All these findings, together, point out that common mechanism respond to this combined stress in different plants and support our interpretation of the present results. In addition, rehydration stress dramatically induced proteolysis-related processes (Figure 5), as well as inhibition of ethylene BP that are related in tolerance to starvation and cellular damage, supporting the notion that rehydration stress leads to NCPP in a similar way as other stresses, such as those triggered by the uncoupling treatments or high energy demands (Establés-Ortiz et al., 2016).

The responses to osmotic stress and ABA were induced during rehydration (Table 1; Figure 5). Deficiency in ABA has been related to increased susceptibility to NCPP (Alferez et al., 2005; Romero et al., 2012). In addition, the previous study has shown that abrupt changes from low to high RH induce sharp changes in water potential and its components, both osmotic and turgor potentials in citrus fruit (Alférez et al., 2010), and this has been related to the induction of PL activities (Cronje et al., 2017) and the interplay between PLs and ABA (Romero et al., 2013a, 2014). In this context, it is noteworthy that we found significantly induced BP of calcium-dependent phospholipid binding during rehydration stress. Many plant phospholipase D (PLDs) contain a Ca^2+^-dependent phospholipid-binding C2 domain (protein kinase C-conserved 2 domain), require Ca^2+^ for activity (Wang, 2000), and have broad substrate specificity, hydrolyzing several common membrane phospholipids, and playing a critical role in many cellular processes, such as signal transduction, membrane trafficking, cytoskeletal rearrangements, and membrane degradation (Qin and Wang, 2002). We also found activation of PLD downstream responses, such as jasmonic acid signaling and cellular periphery (membrane), which together, reinforce the notion of NCPP due to rehydration stress is a result of membrane disorganization *via* phospholipid catabolism. Interestingly, activation of different genes encoding PLD isoforms has been also found to be an early defense response against starvation in citrus fruit; however, prolonged stress may result in higher and constant activation of PLD genes that are also involved in NCPP damage development (Romero et al., 2020b).

A significant number of genes related to response to hypoxia were specifically induced by changing RH conditions (Table 1; Figure 5). The hypoxia conditions triggered by energy failure due to uncoupling and subsequent drop in ATP levels (Alférez et al., 2007) or high energy demand in fruits held at high RH (Establés-Ortiz et al., 2016) have been related to increased NCPP and peel damage in citrus flavedo comparable to NCPP, as stated above. This notion relates tightly with the previous findings by our groups (Establés-Ortiz et al., 2016; Romero et al., 2020a) and others (Rawyler et al., 1999, 2002) describing energy shortage as a mechanism of membrane integrity loss in plants, that in the case of citrus originates NCPP during post-harvest as well (Alférez et al., 2007; Cajuste et al., 2011). In other plants, resistant to water stress exist compensatory mechanisms for membrane integrity upon rehydration tightly regulated by hormones (Dong et al., 2019), but prolonged stress can reduce the ability of the plant to compensate stress, leading to membrane damage. A similar mechanism appears to be operating during NCPP after rehydration in citrus fruit.

A central dogma in citrus post-harvest is that disparate conditions lead to similar peel disorders, as the fruit can only develop a limited number of phenological responses (Grierson, 1986). In this sense, both this study and results from Establés-Ortiz et al. (2016) and Romero et al. (2020a) support that not only response to hypoxia, but also a response to other conditions, such as wounding, oxidative stress, and salicylic acid, or the repression of the regulation of ethylene biosynthesis, among others, were upregulated in response to both energy shortage and rehydration stress, ultimately leading to undistinguishable NCPP symptoms.

## Conclusions

In a nutshell, global transcriptomic responses to rehydration leading to NCPP during post-harvest can be considered as a palimpsest, since the build-up of specific responses to this stress occurs upon ongoing common responses to dehydration, resulting in a complex expression pattern in the fruit. This expression pattern shares many common features with responses to the other stresses. The fact that peel disorders developed in response to these disparate abiotic stresses are similar in morphology, has blurred for years a correct understanding of the causes and has often led to confusion. This study illustrates the way global molecular analysis can help in discriminating between common and unique causes of post-harvest disorders in citrus.

## Data Availability Statement

The datasets presented in this study can be found in online repositories. The names of the repository/repositories and accession number(s) can be found in the https://zenodo.org/record/5171732#.YRDdJkDtZdg.

## Author Contributions

ML and FA conceived the project. PR and FA performed the research and analyzed the data. FA wrote the original draft with contributions by PR and ML. All authors accepted the final manuscript.

## Conflict of Interest

The authors declare that the research was conducted in the absence of any commercial or financial relationships that could be construed as a potential conflict of interest.

## Publisher's Note

All claims expressed in this article are solely those of the authors and do not necessarily represent those of their affiliated organizations, or those of the publisher, the editors and the reviewers. Any product that may be evaluated in this article, or claim that may be made by its manufacturer, is not guaranteed or endorsed by the publisher.

## Supplementary Material

Supplementary Table 1 List of differentially expressed genes (DEG, FDR < 0.01, cutoff Log_2_FC ≥ ±1) when compared the different RH storage conditions vs. the freshly harvested (FH) fruit.

Supplementary Table 2 Biological processes related to NCPP development, rehydration, and dehydration stresses, and fruit detachment and storage, overrepresented in the set of induced (up arrow), and repressed (down arrow) DEG when comparing the indicated storage conditions with respect to freshly harvested (FH) fruit.

Supplementary Table 3 Molecular functions related to NCPP development, rehydration, and dehydration stresses, and fruit detachment and storage, overrepresented in the set of induced (up arrow), and repressed (down arrow) DEG when comparing the indicated storage conditions with respect to freshly harvested (FH) fruit.

Supplementary Table 4 Cellular components related to NCPP development, rehydration, and dehydration stresses, and fruit detachment and storage, overrepresented in the set of induced (up arrow), and repressed (down arrow) DEG when comparing the indicated storage conditions with respect to freshly harvested (FH) fruit.

Supplementary Table 5 Metabolic pathways related to NCPP development, rehydration, and dehydration stresses, and fruit detachment and storage, overrepresented in the set of induced (up arrow), and repressed (down arrow) DEG when comparing the indicated storage conditions with respect to freshly harvested (FH) fruit.
