# Supplementary material for: Differential Transcriptomic Regulation in Sweet Orange Fruit (Citrus sinensis L. Osbeck) Following Dehydration and Rehydration Conditions Leading to Peel Damage
Source: Front Plant Sci. 2021 Aug 31;12:732821. doi: 10.3389/fpls.2021.732821 (PMC8438417; doi:10.3389/fpls.2021.732821)
Supplement: Supplementary Table 2 — Biological processes related to NCPP development, rehydration, and dehydration stresses, and fruit detachment and storage, overrepresented in the set of induced (up arrow), and repressed (down arrow) DEG when comparing the indicated storage conditions with respect to freshly harvested (FH) fruit. [file Table_2.DOCX]

|  |  |  |  |  |  |  |
| --- | --- | --- | --- | --- | --- | --- |
| **Pattern** | **GO ID** | **Biological Process** | **10d 90%** | **10d 30%** | **4d 30%** | **4d 30% +  6d 90%** |
| **1. Specifically related to NCPP development** | | |  |  |  |  |
|  | GO:0044273 | sulfur compound catabolic process |  | ↓ |  | ↑ |
|  | GO:0009269 | response to desiccation | ↓ |  |  | ↑ |
|  | GO:0043090 | amino acid import | ↓ |  |  | ↑ |
|  | GO:0007623 | circadian rhythm |  |  |  | ↑ |
|  | GO:0009411 | response to UV |  |  |  | ↑ |
|  | GO:0019722 | calcium-mediated signaling |  |  |  | ↑ |
|  | GO:0006289 | nucleotide-excision repair |  |  |  | ↓ |
|  | GO:0006857 | oligopeptide transport |  |  |  | ↓ |
|  | GO:0009626 | plant-type hypersensitive response |  |  |  | ↓ |
|  | GO:0010364 | regulation of ethylene biosynthetic process |  |  |  | ↓ |
|  | GO:0045926 | negative regulation of growth |  |  |  | ↓ |
|  | GO:0048281 | inflorescence morphogenesis |  |  |  | ↓ |
|  | GO:0090305 | nucleic acid phosphodiester bond hydrolysis |  |  |  | ↓ |
|  | GO:2000069 | regulation of post-embryonic root development |  |  |  | ↓ |
| **2. Partially related to NCPP development** | | |  |  |  |  |
|  | GO:0009805 | coumarin biosynthetic process | ↓ | ↓ | ↑ | ↑ |
|  | GO:0009835 | fruit ripening |  | ↓ | ↑ | ↑ |
|  | GO:0006879 | cellular iron ion homeostasis | ↓ |  | ↑ | ↑ |
|  | GO:0009696 | salicylic acid metabolic process | ↓ |  | ↑ | ↑ |
|  | GO:0009854 | oxidative photosynthetic carbon pathway | ↓ |  | ↑ | ↑ |
|  | GO:0048564 | photosystem I assembly | ↓ |  | ↑ | ↑ |
|  | GO:0006516 | glycoprotein catabolic process |  |  | ↑ | ↑ |
|  | GO:0009817 | defense response to fungus, incompatible interaction |  |  | ↑ | ↑ |
|  | GO:0009902 | chloroplast relocation |  |  | ↑ | ↑ |
|  | GO:0015689 | molybdate ion transport |  |  | ↑ | ↑ |
|  | GO:0006561 | proline biosynthetic process | ↑ |  | ↓ | ↓ |
|  | GO:0007346 | regulation of mitotic cell cycle | ↑ |  | ↓ | ↓ |
|  | GO:0009750 | response to fructose stimulus | ↑ |  | ↓ | ↓ |
|  | GO:0010252 | auxin homeostasis | ↑ |  | ↓ | ↓ |
|  | GO:0046950 | cellular ketone body metabolic process | ↑ |  | ↓ | ↓ |
|  | GO:0071577 | zinc ion transmembrane transport | ↑ |  | ↓ | ↓ |
|  | GO:0009749 | response to glucose stimulus |  |  | ↓ | ↓ |
|  | GO:0009911 | positive regulation of flower development |  |  | ↓ | ↓ |
|  | GO:0010204 | defense response signaling pathway, resistance gene-independent |  |  | ↓ | ↓ |
|  | GO:0015937 | coenzyme A biosynthetic process |  |  | ↓ | ↓ |
|  | GO:0019058 | viral infectious cycle |  |  | ↓ | ↓ |
|  | GO:0019520 | aldonic acid metabolic process |  |  | ↓ | ↓ |
|  | GO:0046247 | terpene catabolic process |  |  | ↓ | ↓ |
|  | GO:0072530 | purine-containing compound transmembrane transport |  |  | ↓ | ↓ |
| **3. Related to dehydration and rehydration stresses, but not to NCPP development** | | |  |  |  |  |
|  | GO:0008295 | spermidine biosynthetic process |  | ↑ |  | ↑ |
|  | GO:0009739 | response to gibberellin stimulus |  | ↑ |  | ↑ |
|  | GO:0043622 | cortical microtubule organization |  | ↑ |  | ↑ |
|  | GO:0006739 | NADP metabolic process |  | ↓ |  | ↓ |
|  | GO:0019761 | glucosinolate biosynthetic process |  | ↓ |  | ↓ |
| **4. Early responses to dehydration reversed by high RH** | | |  |  |  |  |
|  | GO:0015809 | arginine transport | ↑ |  | ↓ | ↑ |
|  | GO:0016123 | xanthophyll biosynthetic process | ↓ |  | ↑ | ↓ |
| **5. Early and late responses to dehydration stress** | | |  |  |  |  |
|  | GO:0009845 | seed germination | ↓ | ↑ | ↑ | ↑ |
|  | GO:0030026 | cellular manganese ion homeostasis | ↓ | ↑ | ↑ | ↑ |
|  | GO:0042572 | retinol metabolic process | ↓ | ↑ | ↑ | ↑ |
|  | GO:0006011 | UDP-glucose metabolic process |  | ↑ | ↑ | ↑ |
|  | GO:0006776 | vitamin A metabolic process |  | ↑ | ↑ | ↑ |
|  | GO:0009833 | primary cell wall biogenesis |  | ↑ | ↑ | ↑ |
|  | GO:0010189 | vitamin E biosynthetic process |  | ↑ | ↑ | ↑ |
|  | GO:0019252 | starch biosynthetic process |  | ↑ | ↑ | ↑ |
|  | GO:0019253 | reductive pentose-phosphate cycle |  | ↑ | ↑ | ↑ |
|  | GO:0030244 | cellulose biosynthetic process |  | ↑ | ↑ | ↑ |
|  | GO:0051592 | response to calcium ion |  | ↑ | ↑ | ↑ |
|  | GO:0009734 | auxin mediated signaling pathway | ↑ | ↓ | ↓ | ↓ |
|  | GO:0010043 | response to zinc ion | ↑ | ↓ | ↓ | ↓ |
|  | GO:0001887 | selenium compound metabolic process |  | ↓ | ↓ | ↓ |
|  | GO:0006168 | adenine salvage |  | ↓ | ↓ | ↓ |
|  | GO:0006636 | unsaturated fatty acid biosynthetic process |  | ↓ | ↓ | ↓ |
|  | GO:0016115 | terpenoid catabolic process |  | ↓ | ↓ | ↓ |
|  | GO:0019643 | reductive tricarboxylic acid cycle |  | ↓ | ↓ | ↓ |
| **6. Related to detachment, storage or senescence** | | |  |  |  |  |
|  | GO:0010112 | regulation of systemic acquired resistance | ↑ | ↑ |  | ↑ |
|  | GO:0010288 | response to lead ion | ↑ | ↑ |  | ↑ |
|  | GO:0032957 | inositol trisphosphate metabolic process | ↑ | ↑ |  | ↑ |
|  | GO:0048366 | leaf development | ↑ | ↑ |  | ↑ |
|  | GO:0009753 | response to jasmonic acid stimulus | ↑ | ↑ | ↓ | ↑ |
|  | GO:0009970 | cellular response to sulfate starvation | ↓ | ↓ | ↓ | ↓ |
