# Supplementary material for: Differential Transcriptomic Regulation in Sweet Orange Fruit (Citrus sinensis L. Osbeck) Following Dehydration and Rehydration Conditions Leading to Peel Damage
Source: Front Plant Sci. 2021 Aug 31;12:732821. doi: 10.3389/fpls.2021.732821 (PMC8438417; doi:10.3389/fpls.2021.732821)
Supplement: Supplementary Table 3 — Molecular functions related to NCPP development, rehydration, and dehydration stresses, and fruit detachment and storage, overrepresented in the set of induced (up arrow), and repressed (down arrow) DEG when comparing the indicated storage conditions with respect to freshly harvested (FH) fruit. [file Table_3.DOCX]

|  |  |  |  |  |  |  |
| --- | --- | --- | --- | --- | --- | --- |
| **Pattern** | **GO ID** | **Molecular Function** | **10d 90%** | **10d 30%** | **4d 30%** | **4d 30% +  6d 90%** |
| **1. Specifically related to NCPP development** | | |  |  |  |  |
|  | GO:0016597 | amino acid binding |  |  |  | ↑ |
|  | GO:0043225 | anion transmembrane-transporting ATPase activity |  |  |  | ↑ |
|  | GO:0005507 | copper ion binding |  |  |  | ↑ |
|  | GO:0004445 | inositol-polyphosphate 5-phosphatase activity |  |  |  | ↑ |
|  | GO:0016872 | intramolecular lyase activity |  |  |  | ↑ |
|  | GO:0016652 | oxidoreductase activity, acting on NAD(P)H, NAD(P) as acceptor |  |  |  | ↑ |
|  | GO:0016647 | oxidoreductase activity, acting on the CH-NH group of donors, oxygen as acceptor |  |  |  | ↑ |
|  | GO:0008131 | primary amine oxidase activity |  |  |  | ↑ |
|  | GO:0008810 | cellulase activity |  |  |  | ↓ |
|  | GO:0005034 | osmosensor activity |  |  |  | ↓ |
|  | GO:0030674 | protein binding, bridging |  |  |  | ↓ |
|  | GO:0015105 | arsenite transmembrane transporter activity | ↑ |  |  | ↓ |
|  | GO:0050592 | 4-hydroxyphenylacetaldehyde oxime monooxygenase activity |  | ↑ |  | ↓ |
|  | GO:0047085 | hydroxyphenylacetonitrile 2-monooxygenase activity |  | ↑ |  | ↓ |
|  | GO:0015250 | water channel activity |  | ↑ |  | ↓ |
|  | GO:0016630 | protochlorophyllide reductase activity |  |  | ↑ | ↓ |
| **2. Partially related to NCPP development** | | |  |  |  |  |
|  | GO:0003962 | cystathionine gamma-synthase activity |  |  | ↑ | ↑ |
|  | GO:0015098 | molybdate ion transmembrane transporter activity |  |  | ↑ | ↑ |
|  | GO:0045735 | nutrient reservoir activity |  |  | ↑ | ↑ |
|  | GO:0004575 | sucrose alpha-glucosidase activity |  |  | ↑ | ↑ |
|  | GO:0051537 | 2 iron, 2 sulfur cluster binding | ↓ |  | ↑ | ↑ |
|  | GO:0004032 | alditol:NADP+ 1-oxidoreductase activity | ↓ |  | ↑ | ↑ |
|  | GO:0004176 | ATP-dependent peptidase activity | ↓ |  | ↑ | ↑ |
|  | GO:0009882 | blue light photoreceptor activity | ↓ |  | ↑ | ↑ |
|  | GO:0010277 | chlorophyllide a oxygenase [overall] activity | ↓ |  | ↑ | ↑ |
|  | GO:0003904 | deoxyribodipyrimidine photo-lyase activity | ↓ |  | ↑ | ↑ |
|  | GO:0015238 | drug transmembrane transporter activity | ↓ |  | ↑ | ↑ |
|  | GO:0008199 | ferric iron binding | ↓ |  | ↑ | ↑ |
|  | GO:0008465 | glycerate dehydrogenase activity | ↓ |  | ↑ | ↑ |
|  | GO:0010297 | heteropolysaccharide binding | ↓ |  | ↑ | ↑ |
|  | GO:0008266 | poly(U) RNA binding | ↓ |  | ↑ | ↑ |
|  | GO:0080030 | methyl indole-3-acetate esterase activity | ↓ | ↓ | ↑ | ↑ |
|  | GO:0080032 | methyl jasmonate esterase activity | ↓ | ↓ | ↑ | ↑ |
|  | GO:0080031 | methyl salicylate esterase activity | ↓ | ↓ | ↑ | ↑ |
|  | GO:0015215 | nucleotide transmembrane transporter activity |  |  | ↓ | ↓ |
|  | GO:0005102 | receptor binding |  |  | ↓ | ↓ |
|  | GO:0016844 | strictosidine synthase activity |  |  | ↓ | ↓ |
|  | GO:0000976 | transcription regulatory region sequence-specific DNA binding |  |  | ↓ | ↓ |
|  | GO:0005544 | calcium-dependent phospholipid binding | ↑ |  | ↓ | ↓ |
|  | GO:0008061 | chitin binding | ↑ |  | ↓ | ↓ |
|  | GO:0004568 | chitinase activity | ↑ |  | ↓ | ↓ |
|  | GO:0004129 | cytochrome-c oxidase activity | ↑ |  | ↓ | ↓ |
|  | GO:0003939 | L-iditol 2-dehydrogenase activity | ↑ |  | ↓ | ↓ |
|  | GO:0015120 | phosphoglycerate transmembrane transporter activity | ↑ |  | ↓ | ↓ |
|  | GO:0071917 | triose-phosphate transmembrane transporter activity | ↑ |  | ↓ | ↓ |
|  | GO:0005385 | zinc ion transmembrane transporter activity | ↑ |  | ↓ | ↓ |
|  | GO:0047262 | polygalacturonate 4-alpha-galacturonosyltransferase activity | ↑ | ↑ | ↓ | ↓ |
| **3. Related to dehydration and rehydration stresses, but not to NCPP development** | | |  |  |  |  |
|  | GO:0008889 | glycerophosphodiester phosphodiesterase activity |  | ↑ |  | ↑ |
|  | GO:0052692 | raffinose alpha-galactosidase activity |  | ↑ |  | ↑ |
|  | GO:0008515 | sucrose transmembrane transporter activity |  | ↑ |  | ↑ |
|  | GO:0030551 | cyclic nucleotide binding |  | ↑ |  | ↑ |
|  | GO:0000062 | fatty-acyl-CoA binding |  | ↑ |  | ↑ |
|  | GO:0005275 | amine transmembrane transporter activity |  | ↑ | ↓ | ↑ |
|  | GO:0005509 | calcium ion binding |  | ↑ | ↓ | ↑ |
| **4. Early responses to dehydration reversed by high RH** | | |  |  |  |  |
|  | GO:0047980 | hippurate hydrolase activity | ↑ |  |  | ↑ |
|  | GO:0010178 | IAA-amino acid conjugate hydrolase activity | ↑ |  |  | ↑ |
|  | GO:0004715 | non-membrane spanning protein tyrosine kinase activity | ↑ |  |  | ↑ |
| **5. Early and late responses to dehydration stress** | | |  |  |  |  |
|  | GO:0016760 | cellulose synthase (UDP-forming) activity |  | ↑ | ↑ | ↑ |
|  | GO:0004332 | fructose-bisphosphate aldolase activity |  | ↑ | ↑ | ↑ |
|  | GO:0004356 | glutamate-ammonia ligase activity |  | ↑ | ↑ | ↑ |
|  | GO:0004602 | glutathione peroxidase activity |  | ↑ | ↑ | ↑ |
|  | GO:0004365 | glyceraldehyde-3-phosphate dehydrogenase (NAD+) (phosphorylating) activity |  | ↑ | ↑ | ↑ |
|  | GO:0047100 | glyceraldehyde-3-phosphate dehydrogenase (NADP+) (phosphorylating) activity |  | ↑ | ↑ | ↑ |
|  | GO:0004366 | glycerol-3-phosphate O-acyltransferase activity |  | ↑ | ↑ | ↑ |
|  | GO:0080044 | quercetin 7-O-glucosyltransferase activity |  | ↑ | ↑ | ↑ |
|  | GO:0004185 | serine-type carboxypeptidase activity |  | ↑ | ↑ | ↑ |
|  | GO:0009011 | starch synthase activity |  | ↑ | ↑ | ↑ |
|  | GO:0071771 | aldehyde decarbonylase activity | ↓ | ↑ | ↑ | ↑ |
|  | GO:0016168 | chlorophyll binding | ↓ | ↑ | ↑ | ↑ |
|  | GO:0080043 | quercetin 3-O-glucosyltransferase activity | ↓ | ↑ | ↑ | ↑ |
|  | GO:0045549 | 9-cis-epoxycarotenoid dioxygenase activity |  | ↓ | ↓ | ↓ |
|  | GO:0003999 | adenine phosphoribosyltransferase activity |  | ↓ | ↓ | ↓ |
|  | GO:0047763 | caffeate O-methyltransferase activity |  | ↓ | ↓ | ↓ |
|  | GO:0004180 | carboxypeptidase activity |  | ↓ | ↓ | ↓ |
|  | GO:0004450 | isocitrate dehydrogenase (NADP+) activity |  | ↓ | ↓ | ↓ |
|  | GO:0046983 | protein dimerization activity |  | ↓ | ↓ | ↓ |
|  | GO:0010334 | sesquiterpene synthase activity |  | ↓ | ↓ | ↓ |
|  | GO:0004781 | sulfate adenylyltransferase (ATP) activity |  | ↓ | ↓ | ↓ |
| **6. Related to detachment, storage or senescence** | | |  |  |  |  |
|  | GO:0047325 | inositol tetrakisphosphate 1-kinase activity | ↑ | ↑ |  | ↑ |
|  | GO:0052726 | inositol-1,3,4-trisphosphate 5-kinase activity | ↑ | ↑ |  | ↑ |
|  | GO:0052725 | inositol-1,3,4-trisphosphate 6-kinase activity | ↑ | ↑ |  | ↑ |
|  | GO:0005516 | calmodulin binding | ↑ | ↑ | ↓ | ↑ |
|  | GO:0046863 | ribulose-1,5-bisphosphate carboxylase/oxygenase activator activity | ↑ | ↑ | ↑ | ↑ |
