# Supplementary material for: Differential Transcriptomic Regulation in Sweet Orange Fruit (Citrus sinensis L. Osbeck) Following Dehydration and Rehydration Conditions Leading to Peel Damage
Source: Front Plant Sci. 2021 Aug 31;12:732821. doi: 10.3389/fpls.2021.732821 (PMC8438417; doi:10.3389/fpls.2021.732821)
Supplement: Supplementary Table 4 — Cellular components related to NCPP development, rehydration, and dehydration stresses, and fruit detachment and storage, overrepresented in the set of induced (up arrow), and repressed (down arrow) DEG when comparing the indicated storage conditions with respect to freshly harvested (FH) fruit. [file Table_4.DOCX]

|  |  |  |  |  |  |  |
| --- | --- | --- | --- | --- | --- | --- |
| **Pattern** | **GO ID** | **Cellular Component** | **10d 90%** | **10d 30%** | **4d 30%** | **4d 30% + 6d 90%** |
| **1. Specifically related to NCPP development** | | |  |  |  |  |
|  | GO:0017177 | glucosidase II complex |  |  |  | ↑ |
|  | GO:0005887 | integral to plasma membrane |  |  |  | ↑ |
|  | GO:0009523 | photosystem II |  |  |  | ↑ |
|  | GO:0031977 | thylakoid lumen |  |  |  | ↑ |
|  | GO:0030125 | clathrin vesicle coat |  |  |  | ↓ |
| **2. Partially related to NCPP development** | | |  |  |  |  |
|  | GO:0009706 | chloroplast inner membrane | ↓ |  | ↑ | ↑ |
|  | GO:0030093 | chloroplast photosystem I | ↓ |  | ↑ | ↑ |
|  | GO:0009986 | cell surface |  |  | ↓ | ↓ |
|  | GO:0090406 | pollen tube |  |  | ↓ | ↓ |
|  | GO:0045277 | respiratory chain complex IV | ↑ |  | ↓ | ↓ |
| **3. Related to dehydration and rehydration stresses, but not to NCPP development** | | |  |  |  |  |
|  | GO:0005615 | extracellular space |  | ↑ |  | ↑ |
| **4. Early responses to dehydration reversed by high RH** | | |  |  |  |  |
|  | GO:0009543 | chloroplast thylakoid lumen | ↓ |  | ↑ | ↓ |
| **5. Early and late responses to dehydration stress** | | |  |  |  |  |
|  | GO:0009501 | amyloplast |  | ↑ | ↑ | ↑ |
|  | GO:0009505 | plant-type cell wall | ↓ | ↑ | ↑ | ↑ |
|  | GO:0010287 | plastoglobule | ↓ | ↑ | ↑ | ↑ |
|  | GO:0010319 | stromule | ↓ | ↑ | ↑ | ↑ |
|  | GO:0005618 | cell wall |  | ↓ | ↓ | ↓ |
