# Supplementary material for: Differential Transcriptomic Regulation in Sweet Orange Fruit (Citrus sinensis L. Osbeck) Following Dehydration and Rehydration Conditions Leading to Peel Damage
Source: Front Plant Sci. 2021 Aug 31;12:732821. doi: 10.3389/fpls.2021.732821 (PMC8438417; doi:10.3389/fpls.2021.732821)
Supplement: Supplementary Table 5 — Metabolic pathways related to NCPP development, rehydration, and dehydration stresses, and fruit detachment and storage, overrepresented in the set of induced (up arrow), and repressed (down arrow) DEG when comparing the indicated storage conditions with respect to freshly harvested (FH) fruit. [file Table_5.DOCX]

|  |  |  |  |  |  |  |
| --- | --- | --- | --- | --- | --- | --- |
| **Pattern** | **KEGG ID** | **Metabolic Pathway** | **10d 90%** | **10d 30%** | **4d 30%** | **4d 30% +  6d 90%** |
| **1. Specifically related to NCPP development** | | |  |  |  |  |
|  | 562 | Inositol phosphate metabolism |  |  |  | ↑ |
|  | 710 | Carbon fixation in photosynthetic organisms |  |  |  | ↑ |
| **2. Partially related to NCPP development** | | |  |  |  |  |
|  | 650 | Butanoate metabolism |  |  | ↓ | ↓ |
|  | 900 | Terpenoid backbone biosynthesis |  |  | ↓ | ↓ |
| **3. Related to dehydration and rehydration stresses, but not to NCPP development** | | | |  |  |  |
|  | 350 | Tyrosine metabolism |  | ↓ |  | ↓ |
| **5. Early and late responses to dehydration stress** | | |  |  |  |  |
|  | 500 | Starch and sucrose metabolism |  | ↑ | ↑ | ↑ |
|  | 280 | Valine, leucine and isoleucine degradation |  | ↓ | ↓ | ↓ |
|  | 770 | Pantothenate and CoA biosynthesis |  | ↓ | ↓ | ↓ |
| **6. Related to detachment, storage or senescence** | | |  |  |  |  |
|  | 603 | Glycosphingolipid biosynthesis - globo series | ↑ | ↑ |  | ↑ |
|  | 940 | Phenylpropanoid biosynthesis | ↑ | ↑ | ↑ | ↑ |
